# Supplementary material for: A low-cost markerless motion capture system to automate functional gait assessment: Feasibility study
Source: PLoS One. 2026 Jun 2;21(6):e0346606. doi: 10.1371/journal.pone.0346606 (PMC13229360; doi:10.1371/journal.pone.0346606)
Supplement: Attachment — Submitted filename: Darici Cabak Wong 2025 Markerless Response to Reviewers.docx [file pone.0346606.s002.docx]

To the Editor and Reviewers,

We would like to thank the reviewers for their valuable comments. We would in particular like to highlight that both reviewers have identified our lack of clarity with respect to the methods this study, which we have addressed with an additional figure that helps illustrate the processing steps involved in gait extraction and step length and width measurements. We have updated our manuscript substantially based on these comments. Please find our responses to the comments below in red.

**Reviewer 1**

Reviewer #1: 1-Clarify the novelty of the study

Strengthen the Introduction by explicitly distinguishing how your system meaningfully advances existing open-source or low-cost gait analysis tools.

Suggested location: Introduction, paragraph starting at line 64.

We have rewritten the paragraph substantially to better emphasize value in functional gait assessment standardization (paragraph beginning on line 57), as well as the primary gait measures we addressed in this study (step length and width), beginning on line 69.
2-Improve the justification for the sample sizes

Explain why N=3 (validation) and N=5 (FGA experiment) are sufficient for a feasibility study and discuss limitations more explicitly.

Suggested location: Methods – Experiment 1 and Experiment 2.

We acknowledged the low number of subjects as a limitation, discussed the scope of this study and explained our rationale in the paragraph in the Discussion beginning with line 394.

3-Provide clearer participant characteristics

Add demographic details (sex, height, weight, functional ability) to improve transparency and reproducibility.

Suggested location: Methods, lines 167–168.

We have added a table involving the participant information (line 185).

4-Enhance the description of the experimental validation

Provide a clearer explanation of how synchronization between the marker-based and markerless systems was ensured.

Suggested location: Lines 157–164.

We have added a new figure (the new figure 2) about synchronization and provide explanations (lines 157-164 and 201-205).

5-Clarify limitations related to calibration accuracy

Expand on how calibration errors might influence step length, width, and speed measurements in real-world applications.

Suggested location: Discussion, lines 309–315.

In the discussion section we provided information about camera calibration and accuracy (Lines 373-393).

6-Report uncertainty metrics

Include error margins, RMSE, or Bland–Altman plots in addition to R² to better quantify agreement between systems.

Suggested location: Results, Experiment 1.

We updated the figure 3 and included Brand-Altman plots and provided R² and confidence intervals about the measurements (Lines 258 - 274).

7-Strengthen statistical rigor

Even in a feasibility study, add basic statistical comparisons or confidence intervals to reinforce claims of accuracy and consistency.

Suggested location: Lines 223–236.

We reported Brand-Altman plots and provided R² and confidence intervals about the measurements (Lines 258 - 274).

8-Detail the automated FGA scoring algorithm

Provide more transparency on threshold choices, decision boundaries, and any empirical justification.

Suggested location: Lines 189–220.

We provide more clear explanations about the FGA scoring and threshold choices and referred to some papers in the literature (lines 207-232).

9-Clarify how step width and step placement were computed

Present the exact mathematical definitions or formulas used for step width and step length extraction.

Suggested location: Data analysis section (lines 180–187).

We added a new figure (the new figure 2) and illustrated how several parameters including the step width and step length are calculated and provided explanation in the text and figure caption (Lines 165-180).

10-Improve figure readability

Increase resolution, add clearer legends, and use consistent color coding for left/right steps and conditions.

Suggested location: Figures 1–4.

We provided high resolution illustrations of each figure as a .pdf as separate documents within the submission.

11-Expand the discussion on clinical applicability

Discuss practical deployment issues: required space, operator training, setup time, and how it compares with existing clinical workflows.

Suggested location: Discussion, lines 269–278.

In the discussion session, the lines 326-332 and 373-393, we discussed deployment and requirements for the system setup.

12-Include reproducibility information

Provide direct links to the repository, software version, dependencies, and detailed instructions for replicating the setup.

Suggested location: Methods or separate Data Availability section.

In lines 84 to 87, we provided the link to Freemocap software github repository, as well as our data processing pipeline github where code to reproduce the analysis will be made available.

13-Clarify the ability to detect turning and obstacle tasks

Provide a more explicit explanation of how the system handles 3D orientation changes during turning and obstacle negotiation.

Suggested location: Results, Figure 4 and related text.

We provided information about turning, key point detection and potential applications in the lines 364-372 in the discussion section.

14-Improve readability by shortening overly long paragraphs

Break long blocks of text (especially in the Introduction and Discussion) into smaller, thematic paragraphs.

We separated some long paragraphs in the introduction and discussion section.

15-Add future work directions more explicitly

Highlight next steps, including larger cohorts, integration with IMUs, and potential clinical trials.

Suggested location: end of Discussion

In lines 337 to 363 we discussed how the several parameters obtained from our system improve current benefits of FGA. In the lines 364-372, we discussed the applicability of the system to other clinical tests. In the lines 394-402, we mentioned the need to test larger cohorts and finally in the lines 418-428, we discussed the applications with inertial measurement units.

**Reviewer 2**

Major Comments:
1- Sample Size Justification:
The study uses very small sample sizes (N=3 for validation, N=5 for FGA). While this is acceptable for a feasibility study, the authors should explicitly acknowledge this limitation and recommend larger-scale validation in future work. Statistical power and generalizability cannot be inferred from such small groups. Although I know preparing and bringing these types of subjects is very difficult. Therefore, you can mention the difficulty of this process in your manuscript.

We acknowledged the low number of subjects, discussed its limitations and explained our rationale in the discussion paragraph begins with line 394.

2- Technical and Practical Limitations:
The calibration process, while described, may still be a barrier for clinical adoption. The authors should discuss potential simplifications or alternatives for routine clinical use.
The sensitivity to clothing, lighting, and shoe contrast is a practical limitation. Suggestions for standardizing test conditions in clinical environments would strengthen the applicability. This information is very important.

In the discussion section we provided information about camera calibration and accuracy (Lines 373-393). In the lines 403-417, we provided some information about clothing and markerless motion capture systems from the literature and gave own suggestions.

Minor Comments:
The manuscript would benefit from a clearer description of how step width is calculated (the difference in mediolateral foot position between consecutive steps) early in the methods. Although, majority of readers may know.

We added a new figure (currently Figure 2) and illustrated how several parameters including the step width and step length are calculated and provided explanation in the text and figure caption (Lines 165-180).

Recommendation:
Minor Revision. The study presents a promising and innovative approach to automating gait assessment. However, the manuscript requires clarification in methods before it can be considered for publication.

We provide more clear explanations about the FGA scoring and threshold choices and referred to some papers in the literature (lines 207-232).
